# Exploring the predictors of financial impairment in Huntington’s disease using the Enroll-HD dataset

**DOI:** 10.1007/s00415-021-10929-4

**Published:** 2022-02-14

**Authors:** Kate L. Harris, Sarah L. Mason, Roger A. Barker

**Affiliations:** 1 Department of Neurology, University of California San Francisco, San Francisco, United States; 2 Department of Clinical Neurosciences, John Van Geest Centre for Brain Repair, E.D. Adrian Building, Forvie Site, Robinson Way, Cambridge, CB2 0PY UK; 3 MRC-WT Cambridge Stem Cell Institute, University of Cambridge, Cambridge, UK

**Keywords:** Huntington’s disease, Neurodegeneration, Financial impairments, Apathy, Cognitive impairments

## Abstract

**Objectives:**

Huntington’s disease (HD) is a neurodegenerative disease in which cognitive and behavioural symptoms impair the performance of instrumental activities of daily living, including the handling of finances. We sought to determine the prevalence of financial dysfunction in HD, and the demographic and clinical predictors of such impairments.

**Methods:**

We analysed longitudinal data for pre-manifest gene carriers and HD patients from the Enroll-HD dataset. Financial dysfunction was determined by finance-related items in the Total Functional Capacity (TFC) and Functional Assessment (FA) scales. A binary logistical regression model was used to investigate the predictive value of demographic and clinical factors for the development of financial dysfunction.

**Results:**

Financial impairment was found to be common in HD gene carriers, and over half required financial assistance within 5 years from diagnosis. Cognitive impairment, apathy, unemployment and disease severity predicted financial dysfunction in manifest patients. For pre-manifest patients, the predictors were proximity to disease onset and depression.

**Conclusions:**

Loss of financial autonomy is common in HD, and cognitive and psychiatric factors are important in its development. Clinicians must be vigilant to identify patients that may be vulnerable to financial exploitation.

**Supplementary Information:**

The online version contains supplementary material available at 10.1007/s00415-021-10929-4.

## Introduction

Huntington’s disease is a neurodegenerative disease characterised by varying degrees of motor, cognitive and psychiatric dysfunction which together impact an individual’s functional capacity, including instrumental activities of daily living (iADL) such as housekeeping, driving, and managing medications. One important iADL that has been relatively understudied in HD is financial capacity, which refers to the ability to independently manage one’s financial affairs in a manner consistent with one’s personal self-interest [1].

Financial capacity is instrumental for independent living and encompasses a variety of domains including practical skills (e.g., counting coins, making purchases), knowledge of facts (e.g., bank account details) and conceptual understanding (e.g., when making investments and exercising financial judgement). Impairments in these abilities renders an individual at risk for financial insecurity and exploitation [2]. There is a large body of evidence to indicate that financial capacity is diminished in neurodegenerative diseases such as MCI [3–5] and Alzheimer’s Disease (AD) [2, 6], Parkinson’s Disease (PD) [7–9], and frontotemporal dementia (FTD) [10]. Cognitive impairment is an important predictor of financial capacity in these clinical groups [11–15], with difficulties emerging soon after the onset of cognitive decline [16]. Depression also contributes to financial capacity in a non-demented older adult population [17], although the role of depression in financial impairments associated with neurodegeneration is unclear, with some studies showing an association [8, 15, 18], and others not [19–21].

Only a small number of studies have looked to describe financial capacity in HD or understand its underlying factors. Sheppard et al. [22] reported a study of 20 HD patients which showed that HD patients were impaired compared to controls on the Advanced Finances Test, which requires participants to pay three bills, deposit a check, and pay off as much of a credit card balance as possible, whilst ensuring that at least $100 is left in their account. Performance was found to correlate with cognition, but not with depression.

Another study showed that a loss of financial autonomy is one of the earliest functional impairments to occur in HD with approximately 49% of HD gene carriers being unable to manage their finances independently prior to receiving a clinical diagnosis [23].

Despite the limited research in this area, self-reported financial impairments in HD have been widely documented through the inclusion of questions on financial autonomy in the Unified Huntington’s Disease Rating Scale Total Functional Capacity (TFC) and Functional Assessment (FA) sub-scales [24]. Furthermore, these items are included in Enroll-HD (https://enroll-hd.org), a multinational, longitudinal, natural history study that collects demographic and disease-specific information about HD gene carriers. Whilst these items do not give an in-depth understanding of financial capacity, they do provide a measure of “real-world” performance in HD. Understanding the factors which relate to a reduction in financial capacity measured by the TFC and FA could provide a meaningful insight into the reasons why HD patients have difficulty managing their money so early in the disease and potentially identify ways to better support patients with these problems. Therefore, the current study used the Enroll-HD dataset to identify the prevalence of financial impairments in premanifest and manifest gene carriers, and to interrogate the clinical and demographic predictors of these impairments.

## Methods

### Enroll- HD database

This study used the 4th data cut from the Enroll-HD study (https://www.enroll-hd.org), a longitudinal, observational, multinational study of HD gene carriers and controls funded by the CHDI Foundation. As part of the study, participants attend a yearly visit during which they undergo motor, cognitive and psychiatric assessment, and complete self-reported measures of function [25]. The assessments from the protocol that were used in this study are detailed in Table 1.

**Table 1:** Enroll-HD assessments used in the analysis

Scale	Measurement capturing	Items used	Outcome measure
Unified Huntington’s disease rating scale-total motor score [24]	Motor features of HD	All	Total score: 0–124
Unified Huntington’s disease rating scale – total functional capacity	Daily functioning	Item 2: What is your level of functioning: finances	0 = unable, 1 = major assistance, 2 = slight assistance, 3 = normal
Unified Huntington’s disease rating scale – functional assessment	Daily functioning	Item 2: Could the participant manage his/her finances (monthly) without any help	1 = yes or 2 = no
Mini-mental state exam [26]	Global Cognition	All	Total score: 0 – 30 < 24 = dementia cut-off
Symbol digit modality (SDMT) [24]	Processing speed	All	Total number of symbols decoded in 90 s
Verbal fluency (VF) (Animal Naming) [24]	Language/executive function	All	Total animal words generated in 60 s (excluding repetitions or perseverations)
Stroop naming, word, interference [24]	Executive function	All	Total number of blocks verbalised in 45 s for each stage (colour naming, word reading, interference)
Trail-making A and B [27]	Executive function	All	Time take to complete task
Problem behaviour assessment (PBA) – short version	Psychiatric features of HD	Item for depression, irritability and apathy	Frequency: 0–4 0 = never/almost never 1 = seldom (less than once/week) 2 = sometimes (up to 4 times a week) 3 = frequently (most days/5,6 or 7 times a week) 4 = daily/almost daily for most (or all) of day Severity: 0–4 0 = absent 1 = slight, questionable 2 = mild (present, not a problem) 3 = moderate (symptom causing problem) 4 = severe (almost intolerable for carer)

### Selection of participants

Data were available for 20,617 participants comprising of premanifest (pre-HD) and manifest HD gene carriers (mHD) and healthy controls (HC). Participants were excluded from the analysis if they: (1) had not answered the finance-related question in the TFC and FA (2) had a CAG repeat length > 55 due to an increased likelihood of juvenile onset, (3) were under the age of 18 as these individuals were likely to have different financial responsibilities to older participants, (4) had an International Standard Classification of Education (ISCED) of 0 (meaning they had not attended primary school) and (5) had not completed the MMSE. The HD groups (pre-manifest and manifest) therefore were made up of participants with a CAG repeat length between 39 and 55. The healthy control group was created by merging the “gene negative carriers” and “family control” groups from the Enroll-HD dataset. A prevalence analysis was conducted on these groups, and then participants were selected for subsequent analyses based on the criteria outlined below.

#### Demographic and clinical predictors of financial capacity – functional assessment (FA) item

This analysis consisted of manifest HD gene carriers who reported normal financial functioning on the FA at baseline but impaired functioning at a subsequent visit (i.e., scoring 1 = normal and then 0 = impaired, referred to as FAimpaired). These participants were compared to an unimpaired group of manifest gene carriers (FAunimpaired) who reported normal financial management (i.e., scoring “1”) at all visits.

#### Demographic and clinical predictors of financial capacity – total functional capacity (TFC) item

Three groups were created for analysis of the TFC item; those who transitioned from normal functioning to needing slight assistance at some point during the study (scoring “3” then “2”, referred to as “TFCmild”), those who transitioned from needing slight assistance to major assistance (scoring “2” then “1”, referred to as “TFCmoderate”) and those who transitioned from needing major assistance to being unable to manage their finances (scoring “1” then “0”, referred to as “TFCsevere”). We used the same control group for each of these groups, which comprised of manifest patients who scored “3: normal functioning” at all visits (TFCunimpaired). Importantly, this control group was also part of the control group in the FA analysis. However, there was no overlap in the participants included in the impaired groups for the FA or TFC analyses.

Premanifest gene carriers who showed a mild decline in financial management-i.e., from normal functioning (scoring “3”) to needing slight assistance (scoring “2”), were defined as a Pre-HDimpaired group and compared to a control group of premanifest gene carriers who reported “3: normal functioning” at all visits (Pre-HDcontrol).

### Data analysis

#### Prevalence analysis

The prevalence of financial impairments for pre-HD, mHD and HCs was calculated by determining the % of each group who reported financial impairments in the FA or TFC questions at their baseline assessment (i.e., responding “no” on the FA, or < 3 on the TFC). Prevalence was also calculated for manifest gene carriers stratified by disease duration. Chi-square tests were then conducted to assess whether there were significant differences between the groups.

#### Demographic and clinical predictors of financial capacity – FA and TFC analysis

Differences in baseline demographics of control and impaired groups were compared using chi-square tests for nominal data and t tests (FA question) or ANOVA (TFC question) for continuous data. For both questions, the demographic and clinical factors that predicted financial decline were determined using a binary logistic regression, whereby group was the dependent variable, and the following variables were added as covariates: age, sex, disease burden score (DBS), CAG repeat length, ISCED, job class, UHDRS motor score, SDMT, verbal fluency, stroop naming, word, interference, apathy, depression, irritability and MMSE. Sequential regression models were performed until best fit of data was achieved. DBS was calculated using the formula DBS = (CAG_n _− 35.5) × age [28].

## Results

### Prevalence of financial impairment in healthy controls and HD gene carriers

**Fig. 1:**
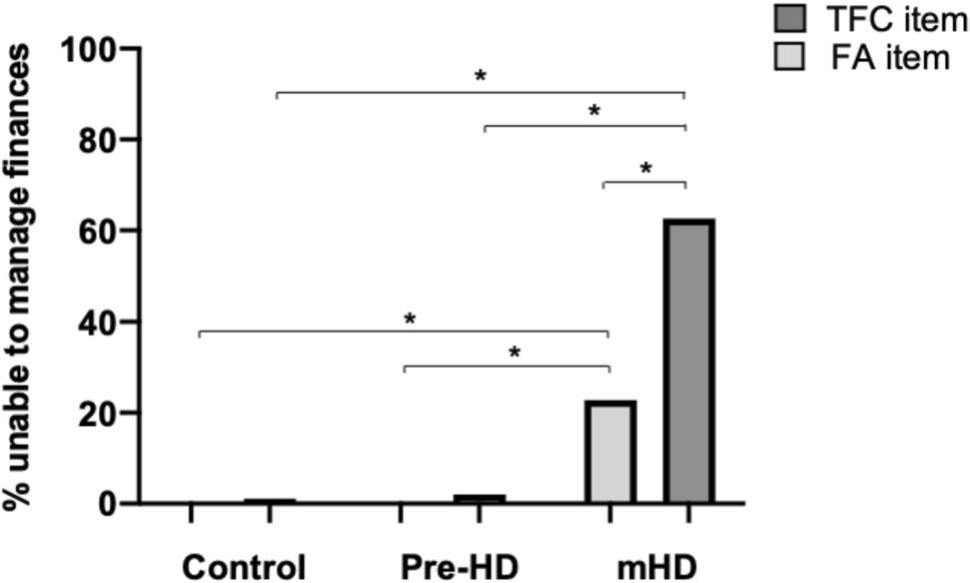
Graph showing the percentage of patients reporting financial impairments on the FA and TFC items. Chi-square tests were used to compare the prevalence of financial impairments across disease groups. Chi-square tests were also used to compare responses on the FA and TFC items. Mean and standard error of the mean are shown. *p < 0.001. FA item: controls *n* = 3715, premanifest *n* = 2806, manifest *n* = 7902. TFC item: controls *n* = 3719, premanifest *n* = 2806, manifest *n* = 8030

A total of 3715 controls, 2806 premanifest and 7902 manifest patients were included in the prevalence analysis. As measured by the FA item, 0.2% of healthy controls and 0.1% of premanifest gene carriers were unable to manage their finances independently on a monthly basis at the baseline visit, whereas 22.8% of manifest gene carriers were unable to do so (χ(1) = 0.341, *p* < 0.001, see Fig. 1). As expected, the prevalence of financial impairment increased with disease duration in manifest patients. For those who had received a diagnosis within the 5 years prior to the baseline visit, 13.8% were unable to manage their finances, rising to 38.8% for those who had been diagnosed for 6–10 years and 55.5% for those who had been diagnosed for greater than 11 years (χ(1) = 0.347, *p* < 0.001, see Fig. 1). In contrast, the TFC financial item yield higher prevalence rates with 1.1% of healthy controls, 2.2% of premanifest patients and 62.6% of manifest patients needing either mild, moderate or major help with finances (χ(1) = 0.635, *p* < 0.001, see Fig. 1). Furthermore, these deficits were evident in 60.4% of those who had been diagnosed for less than 5 years, 79.2% of those diagnosed for 6–10 years and 85% of those diagnosed for greater than 11 years (χ(1) = 0.218 *p* < 0.001).

#### Demographic and clinical predictors of financial capacity – FA analysis

As shown in Table 2, age and CAG were significantly different between the FAimpaired and FAunimpaired groups, although not to a clinically meaningful level. There was no difference between demographic region or marital status (see supplementary tables 1 and 2), and only 10 patients had experienced a change in marital status between visits, so this was unlikely to contribute to any change in financial autonomy. ISCED scores showed a similar distribution of educational levels across groups, although the impaired group were significantly less likely to have an undergraduate degree (see supplementary table 3). The impaired group was also significantly more likely to be unemployed (91.7% vs 66.4%, *p* < 0.001, see Table 2). However, the reasons for unemployment did not differ between the groups, with age-related retirement being the most common reason for both groups (data not shown).

**Table 2:** Demographic information per group

	Manifest HD						Pre-HD	
	FA		TFC				TFC	
	FAunimpaired (*n* = 1179)	FAimpaired (*n* = 339)	TFCunimpaired (*n* = 380)	TFCmild (*n* = 233)	TFCmoderate (*n* = 209)	TFCsevere (*n* = 90)	PreHDcontrol (*n* = 118)	PreHDimpaired (*n* = 31)
Age (years)	52 (11.7)	54.2 (12)*	51.9 (11.4)	50.6 (11.5)	52.7 (11.7)	53.4 (11.5)	41.4 (12.1)	37.3 (10.8)
CAG repeat length	43.3 (2.9)	44 (3.3)***	42.8 (2.5)	43.8 (3)***	44 (3.4)***	43.9 (3.3)***	42 (2.4)	43.8 (3.2)***
DBS	378 (77.8)	441.4 (146.6)***	353.9 (72.5)	406.8 (142.8)***	416.8 (93.2) ***	422.8 (98.7)***	250 (69.7)	288 (83)*
ISCED	3.6 (1.2)	3.4 (1.2)*	3.7 (1.2)	3.5 (1.3)	3.6 (1.2)*	3 (1.2)***	4.2 (1.1)	3.8 (1)
Job class [% unemployed]	3.1 (1.3) [66.4%]	3.8 (0.7)*** [91.7%]	2.6 (1.4) [47.6%]	2.9 (1.3)* [58.8%]	3.6 (0.9)*** [85.2%]	3.9 (0.5)*** [92.8%]	1.8 (1.1)	2.3 (1.4)*
Sex (% female)	50.2%	55.2%	49.5%	44.6%	50.7%	42.7%	65.7%	74.2%
UHDRS motor score	26.3 (13.6)	47 (16.4)***	19.6 (10)	28.3 (12.9)*** †¥	37.1 (14.5) ***‡	43.2 (16.9)***	1.1 (1.4)	2.3 (2)***
SDMT	30.4 (11.4)	16 (8.5)***	35.6 (10.9)	27.3 (10.9)*** †¥	21.1 (9.7)*** †	15.3 (8.2)***	54 (9.4)	46.2 (10.5)***
Verbal Fluency	15.1 (5)	9.8 (4.3)***	16.9 (4.9)	14.1 (5)*** †¥	11.7 (4.7)*** †	9.3 (4.4)***	22.3 (5.8)	20.6 (5.6)
Stroop naming	50.9 (14.7)	32.9 (11.8)***	57.9 (13.7)	47 (14.1)*** †¥	40.3 (12.4)*** †	31.3 (11.6)***	75.9 (14.1)	73.7 (15.9)
Stroop word	67.2 (19.3)	44.2 (15.7)***	76.4 (17.2)	62.6 (19.6)*** †¥	54.4 (17.5)*** †	40.9 (15.2)***	97.7 (16.4)	87.8 (18.1)*
Stroop interference	29 (10.4)	17 (8.6)***	33.6 (9.6)	26.7 (10.1)*** †¥	20.9 (7.9)*** †	16.5 (8.8)***	44.3 (8.9)	42.4 (9.7)
Trail A score	24.9 (1.7)	24.1 (4.1)	24.9 (1.9)	24.9 (1.7) ‡	24.8 (0.2) †	24 (4.3)***	25 (0)	25 (0)
Trail A time	52.5 (30.2)	95.5 (53.6)***	46.9 (25.7)	46.9 (24.7) †¥	72.5 (38.6)*** †	107.8 (59.9)***	25.1 (8.9)	28.1 (10.6)
Trail B score	23.7 (4.3)	17.8 (8.6)***	23.9 (3.8)	24.1 (3.5) †	20.8 (7.1)*** †	16.9 (8.9)***	25 (0)	25 (0)
Trail B time	123.2 (64.5)	193.9 (59.8)***	109 (62)	111.2 (60.8)	171.2 (65.8)***	204 (61.2)***	50.3 (20)	57.5 (24.9)
PBA Irritability	3.1 (4.6)	3.3 (5.1)	2.4 (3.8)	2.9 (4.1)	3.5 (4.8)*	3.1 (4.9)	1.1 (2.2)	4.5 (5.7)***
PBA Apathy	2.5 (3.7)	4.7 (4.7)***	1.6 (3)	2.4 (3.5)*** †¢	3.4 (3.9)*** ‡	4.8 (4.6)***	0.7 (2)	2 (3.2)*
PBA Depression	5.1 (6.3)	5.5 (6.9)	4.5 (6)	4.9 (5.3)	5.8 (6.2)*	5.4 (6)	3.2 (5.3)	9 (8.3)***
MMSE	26.9 (2.6)	23.7 (3.9)***	27.9 (1.9)	26.7 (2.6) †¥	25.4 (2.9)***	23.3 (3.6)***	28.9 (1.3)	28 (2)*

*T* tests were used for the FA and Pre-HD analysis and an ANOVA was used for the TFC analysis. Shows mean and standard deviation in brackets

*DBS* disease burden score, *MMSE* mini-mental state examination, *SDMT* symbol digit modality, *PBA* *problem behaviour assessment (PBA) – short version; ISCED* International standard classification of education

^*^Indicates a significant difference (*p* < 0.05) when compared with controls for each group

^***^Indicates a significant difference (*p* < 0.001) when compared with controls for each group

^†^Indicates a significant difference (*p* < 0.001) when compared with severely impaired patients

^‡^Indicates a significant difference (*p* < 0.05) when compared with severely impaired patients

¥Indicates a significant difference (*p* < 0.001) when compared with moderately impaired patients

¢Indicates a significant difference (*p* < 0.05) when compared with moderately impaired patients

Importantly, the FAimpaired group had a significantly greater DBS score compared to FAunimpaired (see Table 2), suggesting that they were at a more advanced stage of the disease. Indeed, this group exhibited a more severe disease profile, including greater motor and cognitive impairment, alongside a higher degree of reported apathy (see Table 2). The binary logistical regression indicated that DBS, job class, UHDRS motor score, SDMT, verbal fluency, stroop word, MMSE and apathy are all significant predictors of financial impairment in HD (x^2^ (7) = 621.976, *p* < 0.001, see Table 3). Motor scores and apathy were the strongest predictors, with an Exp(B) of 1.044 and 1.086, respectively. The model explained 51.4% (Nagelkerke R^2^) of the variance and correctly classified 85% of cases. Seeing as the impaired group had a significantly lower average MMSE score than the control group (see Table 2), we repeated the analysis including only individuals who scored > 26 on the MMSE, which is a score indicative of normal cognitive function. A logistic regression analysis showed that when MMSE-determined cognitive impairments are controlled for, UHDRS motor score and apathy predict financial impairments in manifest HD (data not shown).

**Table 3:** Logistical regression model of the predictive role of sociodemographic and baseline clinical variables on financial ability as measured by the FA financial item, for the manifest gene carriers

	B	S.E	Wald	df	Sig	Exp(B)
DBS	0.002	0.001	10.132	1	0.001	1.002
Job class	− 0.976	0.323	9.125	1	0.003	0.377
Motor score	0.043	0.006	49.673	1	< 0.001	1.044
SDMT	− 0.076	0.013	35.693	1	< 0.001	0.927
Verbal fluency	− 0.067	0.022	9.059	1	0.003	0.935
MMSE	− 0.071	0.029	6.091	1	0.014	0.932
PBA-Apathy	0.083	0.018	20.495	1	< 0.001	1.086

*DBS* disease burden score, *MMSE* mini-mental state examination, *SDMT* symbol digit modality, *PBA* Problem behaviour assessment (PBA) – short version

FAimpaired* n* = 339*; *FAunimpaired* n* = 1179

#### Demographic and clinical predictors of financial capacity – TFC analysis

The demographic information for these groups is shown in Table 2. There were no differences between the groups in terms of age or sex, and whilst CAG repeat length was statistically different, it is not likely to make a difference clinically. All three impaired groups had a greater motor, cognitive and apathy scores compared to controls (*p* < 0.001), in addition to greater DBS scores (*p* < 0.001) and higher unemployment (*p* > 0.05). Those reporting moderate or severe impairments had a more severe disease profile compared to those reporting milder impairments, as might be expected,

The results of the binary logistic regression analyses for each group are shown in Table 4. For the mildly impaired group, the results indicated that job class, age, CAG repeat length, DBS and MMSE are all significant predictors of financial impairment in HD (x^2^ (6) = 138.736 *p* < 0.001), with the strongest predictor being job class (Exp(B) = 1.724). The model correctly classified 73% of cases and explained 27.6% (Nagelkerke R^2^) of the variance, suggesting that other factors not included in the model also contribute to financial impairment in these patients. For the moderately impaired group, job class, UHDRS motor score, stroop interference and MMSE score were significant predictive factors (x^2^ (4) = 349.129 *p* < 0.001), with employment status being the strongest predictor (Exp(B) = 1.102). This model explained 62% (Nagelkerke R^2^) of the variance and correctly classified 85% of cases. Finally, for the severely impaired group, job class, UHDRS motor score, stroop word, and apathy were the significant predictive factors of financial impairment (x^2^ (4) = 317.794 *p* < 0.001). The model explained 79.2% (Nagelkerke R^2^) of the variance and correctly classified 95% of cases, with apathy being the strongest predictor (Exp(B) = 1.259).

**Table 4:** Logistical regression model of the predictive role of sociodemographic and baseline clinical variables on financial ability as measured by the TFC financial item, for the manifest gene carriers

	B	S.E	Wald	df	Sig	Exp(B)
Mildly impaired						
Age	− 0.108	0.026	17.41	1	< 0.001	0.898
CAG repeat length	− 0.514	0.148	12.113	1	0.001	0.598
Job class	0.544	0.208	6.865	1	0.009	1.724
Motor score	0.059	0.009	41.826	1	< 0.001	1.061
DBS	0.014	0.003	19.442	1	< 0.001	1.014
MMSE	− 0.17	0.044	15.282	1	< 0.001	0.843
Moderately impaired						
Job class	− 1.531	0.342	20.064	1	< 0.001	0.216
Motor score	0.076	0.011	47.303	1	< 0.001	1.079
Stroop interference	− 0.107	0.017	40.472	1	< 0.001	0.899
MMSE	− 0.193	0.056	11.647	1	0.001	0.825
Severely impaired						
Job class	− 4.695	1.064	19.472	1	< 0.001	0.009
Motor score	0.095	0.019	26.545	1	< 0.001	1.1
Stroop word	− 0.109	0.016	45.983	1	< 0.001	0.897
PBA- apathy	0.231	0.056	17	1	< 0.001	1.259

*DBS* disease burden score, *MMSE* mini-mental state examination

TFCunimpaired (*n* = 380); TFCmild (*n* = 233); TFCmoderate (*n* = 209); TFCsevere (*n* = 90)

### Factors that drive financial impairment in premanifest HD

A small group of premanifest gene carriers (*n* = 32, 0.9%) reported a mild decline in financial functioning on the TFC question (from normal functioning to needing slight assistance). Compared to a control group of premanifest gene carriers who reported that they were managing finances normally, these participants had a significantly greater DBS score (*p* < 0.05), and also performed significantly worse on the SDMT (*p* < 0.001) and stroop word tasks (*p* < 0.05, see Table 2). Furthermore, they had significantly greater apathy, irritability, and depression scores, with the latter being particularly high. The binary logistical regression indicated that motor score, SDMT and depression were significant predictors of financial impairment in these participants (x^2^ (3) = 31.780, *p* < 0.001, see Table 5). The model explained 30% (Nagelkerke R^2^) of the variance and correctly classified 82.6% of cases.

**Table 5:** Logistical regression model of the predictive role of sociodemographic and baseline clinical variables on financial ability as measured by the TFC financial item, for the premanifest gene carriers

	B	S.E	Wald	df	Sig	Exp(B)
Motor score	0.327	0.131	6.211	1	0.013	1.387
SDMT	-0.053	0.024	4.790	1	0.029	0.949
Depression	0.103	0.034	9.023	1	0.003	1.108

*SDMT* Symbol digit modalities test

PreHDcontrol (*n* = 118); PreHDimpaired (*n* = 31)

## Discussion

Using a large HD dataset (ENROLL-HD) we found that financial impairment is a significant problem in HD, with 60% of patients reporting that they need assistance with their finances within 5 years of disease onset. We also show that this loss of financial autonomy is associated with specific cognitive and psychiatric impairments in addition to advancing disease and can be seen in premanifest patients approaching disease onset as recorded by DBS.

### Motor impairments

The UHDRS total motor score predicted financial impairment in every financially impaired group included in the study. This differs from the study by Sheppard et al. 2016, which found no correlation between UHDRS total motor score and financial dysfunction in HD, although the sample size of that study may have been too small to detect such a relationship. Importantly, UHDRS total motor score can be considered as a surrogate marker of disease stage in HD, and therefore its significance in the current study may reflect advancing disease as opposed to a direct influence of motor problems. Indeed, disease progression correlates with financial impairment in other neurodegenerative diseases such as AD [2], and in our study DBS was also a predictor of financial impairment. Understanding which specific aspects of disease progression drive impairments in financial capacity in HD is essential and we will consider some of these factors below.

### Cognitive impairments

We found that cognitive dysfunction was associated with a decrease in financial autonomy at all stages of the disease, which is consistent with the wider literature in other neurodegenerative diseases [29] including a previous smaller study in HD [22]. Interestingly, the profile of cognitive deficits was different when looking at financial capacity using the TFC or FA. It remains unclear why this was the case, but one possibility is that the questions were testing different aspects of financial capacity, as discussed in more detail below. Studies in AD have found a correlation between financial impairment and executive function as measured by the trail B test [7, 11, 15], however, performance on this task did not predict financial impairment in the current study. This is perhaps surprising given that executive dysfunction is a cardinal feature of HD [30].

In terms of premanifest gene carriers, the financially impaired group showed a similar cognitive profile to controls, although they did exhibit lower scores on the SDMT and stroop word task, with the former predicting financial impairment. The SDMT has been shown to be an early predictor of disease onset in premanifest HD [31], therefore it could reflect disease onset in this analysis (i.e., with participants showing reduced financial capacity being closer to disease onset than those who remain autonomous). Interestingly, the SDMT is associated with activation in several brain regions known to be affected early in HD such as the caudate nucleus, frontal lobe, insula and posterior cingulate [32], which are also regions implicated in financial decision making [33, 34]. However, future work will be needed to investigate this further.

It is important to note that although the cognitive tasks included in Enroll-HD provide a general measure of cognitive function, they could not be used to ascertain which specific cognitive domains are driving financial impairments in HD (i.e., working memory, attention, arithmetic skills, etc.), and, therefore, this should be a focus of future research.

### Psychiatric impairments

It seems plausible that psychiatric impairments such as depression, apathy and irritability would make it more difficult for HD gene carriers to manage their money independently. However, there have been mixed findings with regard to the role of depression in financial impairments in neurodegenerative diseases [8, 15, 18–21]. In the current study, self-reported apathy was found to be significantly higher in financially impaired manifest patients compared to financially capable controls, whilst irritability and depression scores did not differ between the two groups. Furthermore, apathy predicted financial impairments in patients unable to manage their finances, but not in patients who needed mild or moderate assistance, indicating that apathy may contribute to the later stages of financial decline. A better understanding of this relationship, including the extent to which the apathy at this stage in the disease is a consequence of anti-dopaminergic medication may provide insights into ways in which financial capacity can be managed in manifest HD.

We found that premanifest gene carriers who exhibited financial impairments had higher levels of depression, apathy and irritability compared to controls, with depression predicting such impairments in this small group of individuals. Depression is prevalent in the earlier stages of the HD [35] and in some cases can be responsive to medication such as SSRIs. Going forward it will be important to establish whether treating depression can also reduce the financial impairment experienced in the premanifest stage of HD.

### Employment status

The current study showed that financially impaired patients were significantly more likely to be unemployed than financially capable ones (91.7% vs 66.4%), although it is not possible to deduce the relationship between these two iADLs from the current study. However, it is noteworthy that half of financially impaired patients reported being non-employed due to age-related retirement rather than ill health. Therefore, it is possible that the change of environment associated with retirement had a negative impact on financial management for these individuals but further work will be needed to explore this relationship.

#### Limitations

A major disadvantage of the current study was the lack of detail in the financial questions included in the TFC and FA. Future studies should therefore interrogate which specific aspects of financial management were problematic for HD patients, for example, counting coins, using an ATM, making transfers/investments, etc., [2]. Furthermore, participants were likely to have had differing degrees of financial responsibility from the outset, meaning that financial impairments may be easier to detect in some participants. In addition, there are intrinsic drawbacks of self-report assessments, including a lack of insight, psychiatric symptoms,  and cognitive impairment. We and others have shown that HD patients underestimate motor and cognitive impairments when compared to reports by their companions [36–38], and the same may be the case for financial impairments in the current study. Not every participant in the study will have had a companion to assist with the answering of questions and so their responses may be less accurate than those that did. Finally, whilst we were able to identify some of the factors which contribute to financial dysfunction, we were unable to elucidate the specific aspects of motor or cognitive deficits driving the impairments as the repertoire of test results available to us was limited. This should be a focus for future research.

## Conclusion

The large sample size of the current study has enabled us to estimate the prevalence of financial impairments in HD, which is important given that the ability to manage finances is an essential part of independent living [1]. The fact that almost 1/3 of manifest gene carriers cannot manage their finances renders them at risk of financial insecurity and financial abuse [2]. The results from this study will enable clinicians to recognise potentially vulnerable patients whilst also raising more fundamental questions as to the basis of that deficit and how this can best be managed and/or treated.

## Supplementary Information

Below is the link to the electronic supplementary material.

Supplementary file1 (DOCX 14 KB)

## Data Availability

The Enroll-HD database is available upon request from (https://www.enroll-hd.org).
