# Supplementary material for: Exploring the predictors of financial impairment in Huntington’s disease using the Enroll-HD dataset
Source: J Neurol. 2022 Feb 14;269(7):3501–10. doi: 10.1007/s00415-021-10929-4 (PMC9217841; doi:10.1007/s00415-021-10929-4)
Supplement: Supplementary file 1 — Supplementary file1 (DOCX 14 KB) [file 415_2021_10929_MOESM1_ESM.docx]

**Supplementary**

**Supplementary table 2: Region**

|  | Australasia | Europe | Latin America | Northern America |
| --- | --- | --- | --- | --- |
| FAunimpaired | 5.9% | 60.3% | 0.5% | 33.3% |
| FAImpaired | 3.2% | 63.1% | 1.5% | 32.2% |

**Supplementary table 1: Marital status**

|  | Single | Partnership |
| --- | --- | --- |
| FAunimpaired | 34.2% | 65.8% |
| FAImpaired | 32.7% | 67.3% |

Single includes: Single, divorced, widowed, separated (codes 1,4, 5, 6 in Enroll-HD dataset). Partnership includes: married, partnership (codes 2, 3 in Enroll-HD dataset).

**Supplementary table 3: ISCED**

|  | Primary school | Secondary school | Sixth form/ upper secondary | Work based training | Undergraduate  Degree | PhD doctorate |
| --- | --- | --- | --- | --- | --- | --- |
| FAunimpaired | 2.8% | 15.4% | 32.1% | 17.6% | 29.9% | 2.3% |
| FAImpaired | 5% | 16.8% | 38.1% | 18% | 19.8% | 2.4% |
